# TRIM25 promotes glioblastoma cell growth and invasion via regulation of the PRMT1/c-MYC pathway by targeting the splicing factor NONO

**DOI:** 10.1186/s13046-024-02964-6

**Published:** 2024-02-02

**Authors:** Yike Chen, Xiaohui Xu, Kaikai Ding, Tianchi Tang, Feng Cai, Haocheng Zhang, Zihang Chen, Yangjian Qi, Zaixiang Fu, Ganggui Zhu, Zhangqi Dou, Jinfang Xu, Gao Chen, Qun Wu, Jianxiong Ji, Jianmin Zhang

**Affiliations:** 1 Department of Neurosurgery, Second Affiliated Hospital, School of Medicine, Zhejiang University, Hangzhou, 310000 Zhejiang P. R. China; 2 Key Laboratory of Precise Treatment and Clinical Translational Research of Neurological Diseases, Hangzhou, 310000 Zhejiang P. R. China; 3 Brain Research Institute, Zhejiang University, Hangzhou, 310000 Zhejiang P. R. China; 4 MOE Frontier Science Center for Brain Science & Brain-Machine Integration Zhejiang University, Hangzhou, 310000 Zhejiang P. R. China; 5 Department of Radiation Oncology, the First Affiliated Hospital, School of Medicine, Zhejiang University, Hangzhou, 310000 Zhejiang P. R. China

**Keywords:** Ubiquitination, TRIM25, NONO, Splicing efficiency, c-MYC pathway

## Abstract

**Background:**

Ubiquitination plays an important role in proliferating and invasive characteristic of glioblastoma (GBM), similar to many other cancers. Tripartite motif 25 (TRIM25) is a member of the TRIM family of proteins, which are involved in tumorigenesis through substrate ubiquitination.

**Methods:**

Difference in TRIM25 expression levels between nonneoplastic brain tissue samples and primary glioma samples was demonstrated using publicly available glioblastoma database, immunohistochemistry, and western blotting. TRIM25 knockdown GBM cell lines (LN229 and U251) and patient derived GBM stem-like cells (GSCs) GBM#021 were used to investigate the function of TRIM25 in vivo and in vitro. Co-immunoprecipitation (Co-IP) and mass spectrometry analysis were performed to identify NONO as a protein that interacts with TRIM25. The molecular mechanisms underlying the promotion of GBM development by TRIM25 through NONO were investigated by RNA-seq and validated by qRT-PCR and western blotting.

**Results:**

We observed upregulation of TRIM25 in GBM, correlating with enhanced glioblastoma cell growth and invasion, both in vitro and in vivo. Subsequently, we screened a panel of proteins interacting with TRIM25; mass spectrometry and co-immunoprecipitation revealed that NONO was a potential substrate of TRIM25. TRIM25 knockdown reduced the K63-linked ubiquitination of NONO, thereby suppressing the splicing function of NONO. Dysfunctional NONO resulted in the retention of the second intron in the pre-mRNA of PRMT1, inhibiting the activation of the PRMT1/c-MYC pathway.

**Conclusions:**

Our study demonstrates that TRIM25 promotes glioblastoma cell growth and invasion by regulating the PRMT1/c-MYC pathway through mediation of the splicing factor NONO. Targeting the E3 ligase activity of TRIM25 or the complex interactions between TRIM25 and NONO may prove beneficial in the treatment of GBM.

**Supplementary Information:**

The online version contains supplementary material available at 10.1186/s13046-024-02964-6.

## Background

Glioblastoma multiforme (GBM) is the most common malignant primary brain tumor in adults [1]. Despite substantial progress in surgery, radiotherapy, and chemotherapy, aggressive growth and easy recurrence remain the most prominent features of GBM; resulting in a median survival is 14 to 20 months for affected patients [2–5]. The infiltration of tumor cells into normal tissues significantly contributes to the poor prognosis associated with GBM [6–8]. In the past several years, many researchers have explored the biological changes underlying GBM onset [9, 10], but further efforts are needed to identify effective therapeutic targets for GBM.

Among the various regulatory pathways in human cells, ubiquitination is the most common (and possibly the most important) type of post-translational modification involved in the regulation of many diseases, including cancers [11–13]. Ubiquitination usually results in the formation of a bond between one or more ubiquitin molecules and lysine residues on target proteins [14]. Depending on the type of polyubiquitin chain involved, ubiquitination can have unique functions: K48-linked ubiquitin chains facilitate proteasomal degradation by the 26 S proteasome [14], while K63-linked ubiquitin chains regulate diverse cellular processes (e.g., DNA repair, signal transduction, kinase activity, and endocytosis) [15, 16]. Ubiquitination involves three enzymes, with E3 ubiquitin ligases playing an important role in this process. Consequently, they are implicated in the pathogenesis of many diseases, making them potential targets for treatment [17–19].

Tripartite motif (TRIM) proteins are a subfamily of RING-type E3 ubiquitin ligases. TRIM25 is a member of the TRIM family of proteins, which contain an N-terminal conserved RING domain, two zinc-finger domains known as B-boxes, a coiled-coil region, and a C-terminal PRYSPRY domain [20]. In recent years, the antiviral capabilities of TRIM25 via regulation of the cytosolic retinoic acid-inducible gene I (RIG-I) signaling pathway have been elucidated [21–23]. Recent researches indicate that TRIM25 participates in the development of many tumors [24–28], and there is growing evidence supporting TRIM25 as a potential therapeutic target for tumors. Our study revealed an elevated expression of TRIM25 in GBM. Furthermore, we discovered that TRIM25 mediated the protein arginine methyltransferase 1 (PRMT1)/c-MYC pathway by regulating K63-linked ubiquitination of the oncogene NONO, thereby promoting proliferation and invasion of GBM cells.

## Materials and methods

### Cell culture, transient transfection and lentiviral infection

Human glioblastoma cell lines (U251 and LN229) and the human embryonic kidney cell line 293 (HEK293) were purchased from the iCell Bioscience Inc (Shanghai, China). Patient derived glioblastoma stem-like cells (GSCs) GBM#021 were previously isolated and characterized from surgical specimens of glioblastoma. LN229 and U251 cells were cultured in Dulbecco’s Modified Eagle’s medium (DMEM; Thermo Fisher Scientific; Waltham, MA, USA) supplemented with 10% fetal bovine serum (FBS; Thermo Fisher Scientific). GBM#021 was cultured in Dulbecco’s Modified Eagle Medium/Nutrient Mixture F-12 (DMEM/F-12; Thermo Fisher Scientific) supplemented with 1% B27 (Thermo Fisher Scientific), 1% GlutaMAX (Thermo Fisher Scientific), EGF (20 ng/mL; PeproTech; East Windsor, NJ, USA), and basic FGF (20 ng/mL; PeproTech). Cells were treated with MG132 (20 µM; MedChemExpress; Beijing, China) for 6–8 h to inhibit proteasomal mediated degradation. Cells were maintained at 37 °C in a humidified chamber containing 5% CO2.

Transient transfections for siRNAs were conducted with Lipofectamine 2000 (Thermo Fisher Scientific) according to the following steps: 1 × 10^6^ cells were plated in a 6-well dish. 250 µl of Opti-MEM Medium (Thermo Fisher Scientific) was added to each of two new 1.5 ml Eppendorf tubes, labeled as A and B. In tube A, 5 µl of Lipofectamine 2000 was added, while 6 µl of siRNA was added to tube B. After a 5-minute incubation at room temperature, the entire content of tube A was transferred to tube B. Following the washing of cells with PBS, 1 ml of Opti-MEM Medium was added, and the prepared mixture (500 µl) was gently added to the cells. The cells were then incubated for 12 h at 37 °C before switching back to DMEM complete medium. Transient transfections with plasmids were conducted with Lipofectamine 3000 (Thermo Fisher Scientific) according to the following steps: 1 × 10^6^ cells were plated in a 6-well dish. 250 µl of Opti-MEM Medium (Thermo Fisher Scientific) was added to each of two new 1.5 ml Eppendorf tubes, labeled as A and B. In tube A, 6 µl of Lipofectamine 3000 was added. In tube B, 2 µg of plasmid was added, followed by a 1-minute incubation at room temperature. Additionally, 5 µl of P3000 was added to tube B. After a 5-minute incubation at room temperature, the entire content of tube A was transferred to tube B, with an additional 15-minute incubation at room temperature. Following the washing of cells with PBS, 1 ml of Opti-MEM Medium was added, and the prepared mixture (500 µl) was gently added to the cells. The cells were then incubated for 12 h at 37 °C before switching back to DMEM complete medium.

For establishment of stable TRIM25-knockdown cells, LN229, U251, and GBM#021 were transfected with lentivirus containing two different shRNAs targeting TRIM25 (sh-TRIM25-1, sh-TRIM25-2; OBiO Technology; Shanghai, China). After 48 h, cells were harvest and cultured in DMEM or DMEM/F-12 containing puromycin (2 µg/mL; Thermo Fisher Scientific) for an additional 2 weeks to select for cells containing the constructs. The plasmids and sequences of shRNAs and siRNAs used are listed in Supplementary Table. S1 and S2.

### Immunohistochemistry (IHC), and Western blotting (WB)

IHC, and WB were performed as described previously [29]. The simplified IHC procedure is as follows: The paraffin-embedded sections were deparaffinized and rehydrated, followed by antigen retrieval in 0.01 M citrate buffer at 95 °C for 20 min. After blocking, the sections were incubated with the primary antibody overnight at 4 °C. On the next day, the sections were washed with PBS and incubated with the secondary antibody at room temperature. Visualization is achieved using 3,3’-diaminobenzidine (DAB; Solarbio; Beijing, China) as the substrate for color development, followed by counterstaining with hematoxylin (Beyotime; Haimen, China).The IHC-stained samples were reviewed and evaluated by two blinded pathologists. IHC results were scored according to the following criteria: (1) staining level 0–3: 0, no staining; 1, weak staining; 2, moderate staining; 3, strong staining; (2) percentage of positive cells 0–3: 0, 0%; 1, 1-33%; 2, 34-66%; 3, more than 67%. The sum of these two parts constitutes the final score.

The simplified western blotting procedure is as follows: Cells and tissues were incubated in RIPA buffer (Beyotime) containing a protease and phosphatase inhibitor cocktail (Thermo Fisher Scientific) at 4 °C for 30 min on a shaker. Following centrifugation, quantification, and denaturation, 20 µg of protein were separated using a 10% polyacrylamide gel and transferred to polyvinylidene difluoride (PVDF) membranes (Merck Millipore; Shanghai, China). The membranes were blocked with Tris-Buffered Saline with Tween 20 (TBST) containing 5% skim milk and incubated overnight at 4 °C with the primary antibody. The next day, after washing the membrane with TBST (three times, each for 10 min), incubate it with the secondary antibody conjugated to horseradish peroxidase (HRP) at room temperature for 1 h. Following additional washing, the membrane was exposed using the Bio-Rad Gel Imaging System (Bio-Rad; Irvine, CA, USA). All antibodies used are listed in Supplementary Table. S3.

### Real-time quantitative RT-PCR (qRT-PCR)

Total RNA was extracted from cells using RNA Extraction Kit (Servicebio; Wuhan, China). Reverse transcription (RT) was performed with 0.5–1 µg of total RNA using the SweScript RT I First Strand cDNA Synthesis Kit (Servicebio) according to the manufacturer’s protocols. Quantitative PCR was performed using the SYBR Green qPCR Master Mix (Servicebio) on the Real-Time PCR Detection System (CFX Connect, Bio-Rad; Hercules, CA, USA). GAPDH served as the internal control. Primers used for PCR are listed in Supplementary Table. S4.

### Co-immunoprecipitation (Co-IP) and mass spectrometry analysis

Cells were lysed in IP lysis buffer (Thermo Fisher Scientific) containing protease and phosphatase inhibitor cocktail (Thermo Fisher Scientific). Subsequently, the lysate was quantified to 1 µg/µL and placed on ice for later use. 30 µL of protein A/G magnetic beads (Thermo Fisher Scientific) were added to a 1.5 mL Eppendorf tube and placed on the magnet (DynaMag Magnet; Thermo Fisher Scientific), followed by discarding the supernatant. After repeated washing of the magnetic beads, primary antibodies (1–10 µg) or IgG (1–10 µg) diluted in 200 µL IP lysis buffer were added to the prepared beads, and the mixture was incubated with rotation for 2 h at room temperature. The supernatant of the magnetic bead-Ab complex was discarded, and the lysate (200 µg) was incubated with the magnetic bead-Ab complex overnight at 4 °C with gentle rotation. The processed magnetic bead-Ab-Ag complex underwent immunoblotting or mass spectrometry analysis (PTM BIO; Hangzhou, China). The resulting MS/MS data were processed using MaxQuant search engine (v.1.6.15.0). For the ubiquitination assay, cells were treated with 20 µM MG132 for 6 h before lysis.

### EdU assay

Cell proliferation was measured with an EdU assay kit (Ribo-Bio; Suzhou, China). Experiments were performed according to the kit’s instructions. The representative images were recorded using a Leica inverted fluorescence microscope.

### Cell number counting

The transfected cells (5.0 × 10^4^/well) were seeded into six-well plates. Cells were harvested through trypsinization and counted every 24 h for 3 days. Experiments were performed in triplicate.

### Colony forming assay

The transfected cells (250/well) were seeded into six-well plates and cultured for an additional 2 weeks. After discarding supernatant and washing with PBS, cells were fixed with 4% paraformaldehyde (Solarbio; Beijing, China) and stained with 5% crystal violet (Solarbio). Experiments were performed in triplicate.

### Cell migration and invasion assay

Cell migration and invasion assays were performed in uncoated and Matrigel-coated (TheWell Bioscience; NJ, USA) Transwell chambers (pore size: 8 μm; Corning Costar; NY, USA). Cells (2.5 × 10^4^) and 100 µL medium containing 1% FBS were added to the upper chamber and 600 µL medium containing 30% FBS was added to the lower chamber. After incubating at 37 °C for 24–36 h, migrated or invaded cells were fixed with paraformaldehyde and stained with crystal violet for 15 min. Images were obtained from random fields in each well. Experiments were performed in triplicate.

For the 3D tumor spheroid invasion assay, transfected GBM#021 cells (3 × 10^3^) were seeded on low adherence 96-well plates (Corning Costar) and cultured at 37 °C in a tissue culture incubator for 72 h to promote spheroid formation. 50 µL invasion gel (R&D Systems; MN, USA) was added to each well, and the plates were incubated at 37 °C for 3 days. Images of tumor spheroid were captured every 24 h.

### Animal studies

Athymic nude mice (female, 4-week old; GemPharmatech, Jiangsu, China) were randomly assigned to groups of six animals each. To establish an orthotopic glioma model, we selected LN229 transfected with sh-NC-luciferase (Luc) /sh-TRIM25-2-luciferase (Luc), U251 with sh-NC-luciferase (Luc) /sh-TRIM25-1-luciferase (Luc), and GBM#021 with sh-NC-luciferase (Luc) /sh-TRIM25-2-luciferase (Luc) based on the knockdown efficiency of TRIM25. These cells (5 × 10^5^ cells suspended in 10µL, PBS) were then implanted into the frontal lobes of nude mice using a stereotaxic apparatus (68,001, RWD, Shenzhen, China). Tumor growth was observed by bioluminescence imaging (IVIS spectrum in vivo imaging system, PerkinElmer; Hopkinton, MA, USA) at 7, 14, and 28 days after implantation of transfected LN229 and U251, and 5, 10, and 20 days after implantation of transfected GBM#021, respectively. Animals were humanely sacrificed by cervical dislocation upon exhibiting any symptoms of continuous discomfort, such as severe hunchback posture, decreased activity, apathy, dragging legs, or more than 20% weight loss.

### RNA-Seq and splicing efficiency analysis

Total RNA was extracted from transfected LN229 cells using TRIzol (Thermo Fisher Scientific) and subjected to RNA-Seq. rMATS was used to analyze the different AS events between the control and sh-TRIM25 groups. The method for splicing efficiency calculation was as followed: Efficiency 5’ or 3’= transread count / 5’ or 3’ intron end first base coverage. The splicing efficiency of genes was calculated in R package Splicing Efficiency Analysis and Annotation (SEAA). Visualization was performed using Integrative Genomics Viewer (IGV). The results of RNA-Seq have been uploaded to the GEO database (GSE236661).

### Database and gene enrichment analysis

All datasets were from the following public websites: The Cancer Genome Atlas (TCGA) (https://cancergenome.nih.gov/) and The Chinese Glioma Genome Atlas (CGGA) (http://www.cgga.org.cn/). Using GlioVis (http://gliovis.bioinfo.cnio.es/), a website designed for online analysis of data in the publicly available glioblastoma database, enables gene expression and survival analysis.

### Statistical analysis

All experiments were performed in at least three independent biological replicates and reported as the mean ± the standard error of the mean. The statistical significance was calculated utilizing an unpaired two-tailed Student’s t test for direct comparisons and ANOVA for multigroup comparisons. Survival curves were estimated using the Kaplan–Meier method and compared using the log-rank test. Statistical analysis was conducted using GraphPad Prism version 8.00 software for Windows (GraphPad). *P* values < 0.05 were considered to be statistically significant.

### Ethics approval

All primary glioma tissue samples (*n* = 48) and nonneoplastic brain tissue samples (NBT; *n* = 8) were obtained from the Department of Neurosurgery of The Second Affiliated Hospital of Zhejiang University (SAHZU). All experiments of human tissues and animals were approved by the Research Ethics Committee of SAHZU.

## Results

### TRIM25 is upregulated in GBM and associated with poor prognosis

**Fig. 1:**
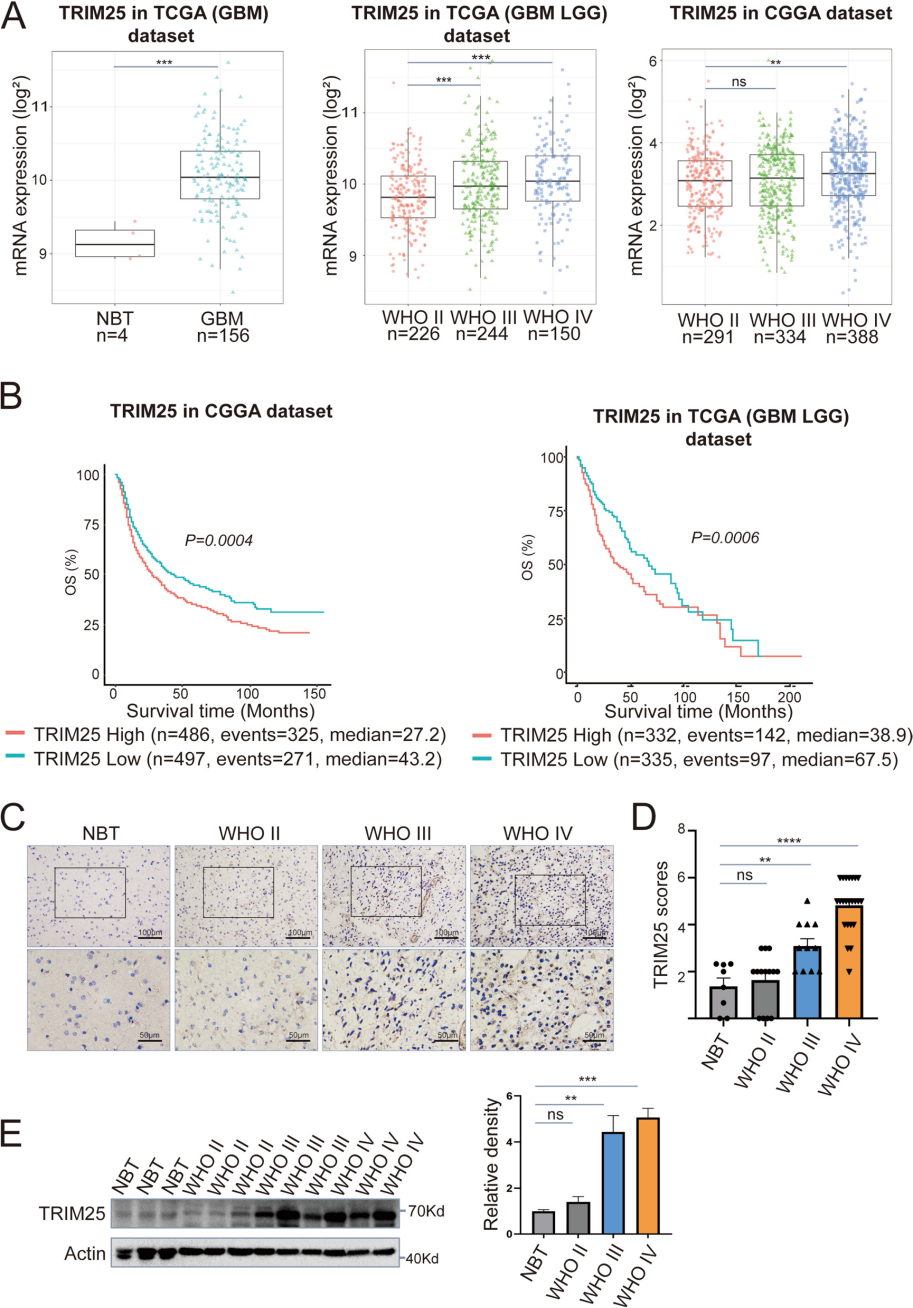
TRIM25 is upregulated in glioma and associated with poor prognosis. **A** TRIM25 mRNA levels in non-tumor tissues (*n* = 4) and GBM tissues (*n* = 156) from TCGA (GBM) datasets (left panel); in WHO grade II (*n* = 226), WHO grade III (*n* = 244), and WHO grade IV (*n* = 150) glioma tissues from TCGA (LGG/GBM) datasets (middle panel); and in WHO grade II (*n* = 291), WHO grade III (*n* = 334), and WHO grade IV (*n* = 388) glioma tissues from CGGA datasets (right panel). **B** Overall survival (OS) of TRIM25 mRNA-high and TRIM25 mRNA-low patients with glioma in CGGA (*n* = 983) and TCGA (*n* = 667) datasets. **C** Representative images of TRIM25 IHC performed on WHO grade II–IV glioma tissues and nonneoplastic brain tissues (NBTs). Scale bar = 100 μm (top) or 50 μm (bottom). **D** Graphical representation of scoring performed for IHC staining of TRIM25 in primary glioma tissues and NBTs. **E** Western blotting analysis of TRIM25 protein levels in primary glioma tissues (*n* = 9) and NBTs (*n* = 3). ns: not significant, ∗: *p* < 0.05, ∗∗: *p* < 0.01, ∗∗∗: *p* < 0.001, and ∗∗∗∗: *p* < 0.0001

To investigate the function of TRIM25 in patients with GBM, we used online tools to examine the mRNA levels of TRIM25 and assess its impact on prognosis in GBM with public data from TCGA and CGGA. The results showed that the TRIM25 expression level was significantly higher in glioblastoma (GBM, *n* = 156) than in NBT (*n* = 4). Furthermore, it was also significantly higher in high-grade glioma than in low-grade glioma (TCGA-LGG GBM, *n* = 620, CGGA, *n* = 1013) (Fig. 1A). Kaplan–Meier survival analysis revealed that patients with glioma expressing high levels of TRIM25 exhibited worse overall survival (OS) compared to patients with lower TRIM25 expression levels (Fig. 1B). To determine whether protein levels were altered, we used IHC to assess TRIM25 protein expression levels in primary glioma samples and NBT samples. The histological grade distribution of the samples included NBT (*n* = 8), World Health Organization (WHO) grade II (*n* = 14), WHO grade III (*n* = 11), and WHO grade IV (*n* = 23). TRIM25 expression was significantly higher in high-grade gliomas than in low-grade gliomas (Fig. 1C, D, Supplementary Fig. S1A), while expression was minimal in NBT samples (Fig. 1C, D, Supplementary Fig. S1A). Western blotting analysis of lysates from 19 primary tumors (WHO grades II-IV) and 4 NBT samples further confirmed the upregulation of TRIM25 in human gliomas (Fig. 1E, Supplementary Fig. S1B). In summary, TRIM25 expression was significantly higher in high-grade gliomas than in low-grade gliomas, suggesting that TRIM25 has potential as a prognostic marker in GBM.

### TRIM25 knockdown inhibits GBM proliferation, migration, and invasion in vitro and in vivo

**Fig. 2:**
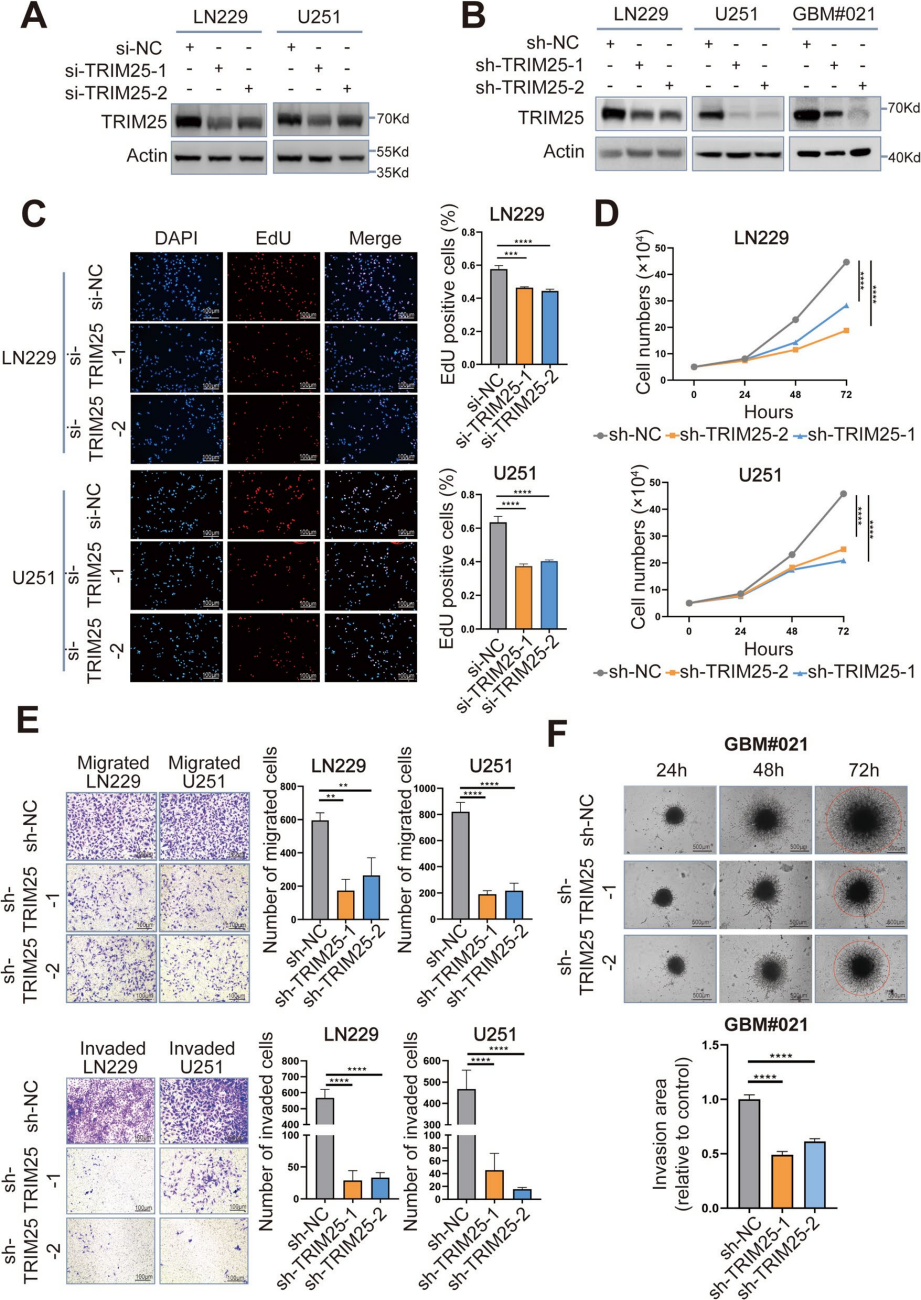
TRIM25 knockdown inhibits GBM proliferation, migration, and invasion in vitro. **A** Western blotting analysis was used to assess TRIM25 knockdown efficiency in LN229 and U251 cells transfected with TRIM25 siRNAs. **B** Western blotting analysis was used to assess TRIM25 knockdown efficiency in LN229, U251 and patient-derived GBM#021 cells transfected with TRIM25 shRNAs. **C** EdU assays of transfected LN229 and U251 cells. Graphical representation of EdU-positive cells in cultures of LN229- and U251-si-NC, si-TRIM25-1, and si-TRIM25-2 cells. Scale bars = 100 μm. **D** Growth curves for TRIM25-knockdown LN229 and U251 cells generated by cell counting over 72 h. **E** Representative images of fixed and stained Transwell migration and invasion assays involving transfected LN229 and U251 cells. Scale bars = 100 μm. **F** Representative images of 3D tumor spheroid invasion assays and quantification of invasion area. Scale bars = 500 μm. ns: not significant, ∗: *p* < 0.05, ∗∗: *p* < 0.01, ∗∗∗: *p* < 0.001, and ∗∗∗∗: *p* < 0.0001

To investigate the role of TRIM25 in GBM, we knocked down TRIM25 expression in LN229 and U251 human glioma cells using two different siRNAs. The efficiency of TRIM25 knockdown was validated by Western blotting (Fig. 2A). The EdU assays showed a significant reduction in cell proliferation in both knockdown groups (TRIM25 siRNA-1 and − 2) (Fig. 2C). To validate the results, cells were transfected with lentiviruses expressing TRIM25 shRNA-1, TRIM25 shRNA-2, or the negative control shRNA (Fig. 2B). At 72 h after cell plating, the cell numbers were significantly lower in knockdown groups compared with control groups (Fig. 2D), indicating that TRIM25 knockdown significantly attenuated the proliferation of LN229 and U251 cells. Colony formation assays further demonstrated the role of TRIM25 in promoting tumor proliferation (Supplementary Fig. S2A). Subsequently, transwell assays were conducted using TRIM25-knockdown LN229 and U251 cells. As illustrated in Fig. 2E, the numbers of invading/migrating cells were significantly lower in both TRIM25-knockdown cell lines compared with control cells.

We then conducted TRIM25 knockdown in patient-derived GBM#021 cells using TRIM25 shRNAs (Fig. 2B). After 72 h of cell plating, the cell numbers were markedly lower in the knockdown groups compared to the control groups (Supplementary Fig. S2B), indicating a significant reduction in the proliferation of GBM#021 cells upon TRIM25 knockdown. Additionally, we assessed the invasive capabilities of TRIM25-knockdown GBM#021 cells through a 3D tumor spheroid invasion assay. The invasion area of TRIM25-knockdown GBM#021 spheroids showed a significantly decrease compared to the controls (Fig. 2F). These results indicated that TRIM25 deficiency in GBM cells leads to crucial impairments in both proliferation and invasion.

**Fig. 3:**
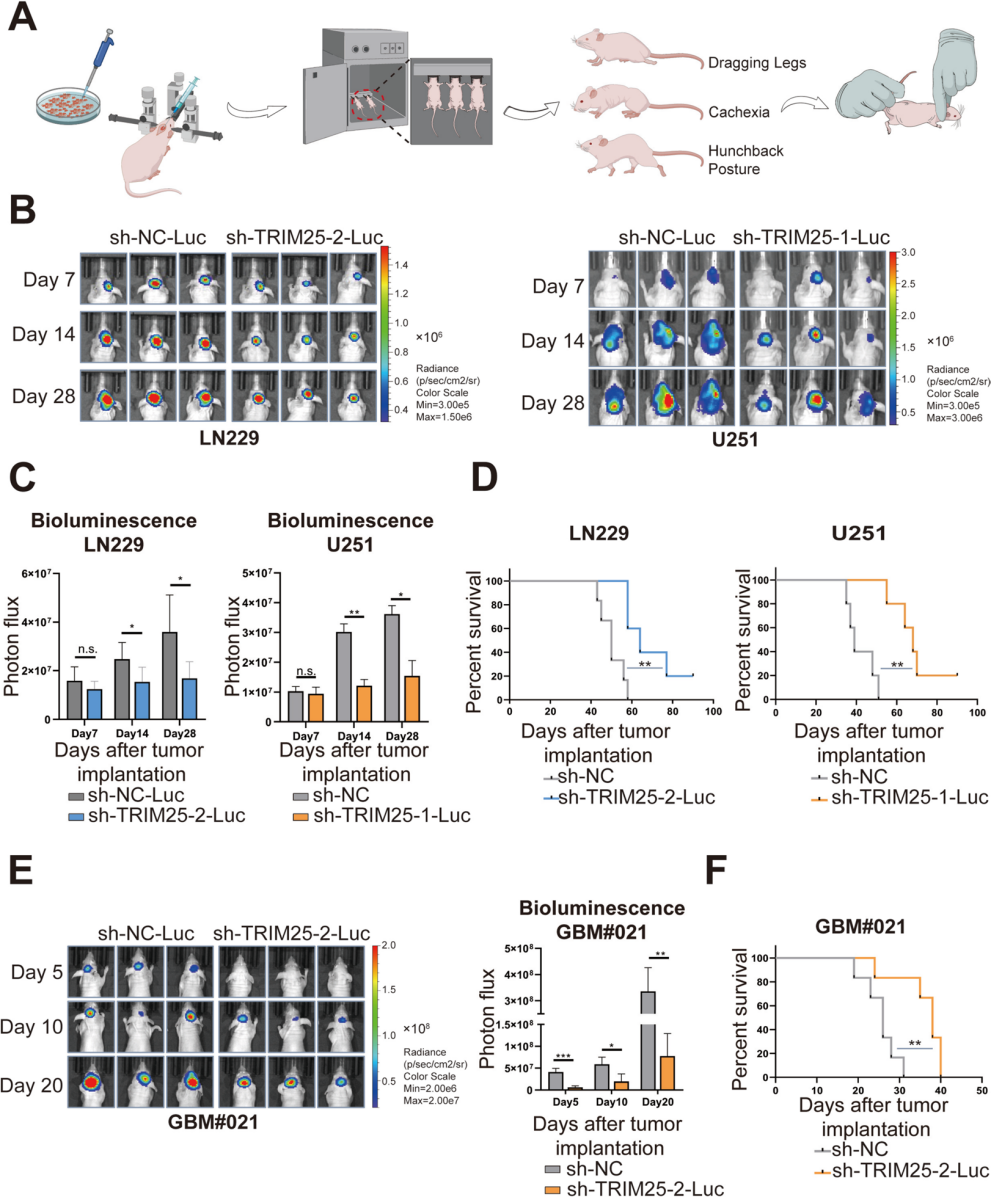
TRIM25 knockdown inhibits GBM cell growth in vivo. **A** In vivo experimental workflow: **a** 5 × 10^5^ transfected glioma cells were harvested from cell culture dishes and implanted into the frontal lobes of nude mice. **b** In vivo bioluminescence imaging of the xenograft models was performed on days 5/7, 10/14, and 20/28 after cell implantation (the numbers of mice subjected to in vivo bioluminescence imaging were as follows: LN229, 6 for sh-NC-Luc and 5 for sh-TRIM25-2-Luc; U251, 5 for sh-NC-Luc and 5 for sh-TRIM25-1-Luc; and GBM#021, 6 for sh-NC-Luc and 6 for sh-TRIM25-2-Luc). **c** Animals were humanely euthanized by cervical dislocation when they showed signs of continuous discomfort, such as severe hunchback posture, decreased activity, apathy, leg dragging, or > 20% weight loss. **B**, **C** Images and quantification of in vivo bioluminescence in transfected LN229 and U251 cell-derived xenografts at indicated times. **D** Kaplan–Meier survival analysis performed with survival data from indicated groups. **E **Images and quantification of in vivo bioluminescence in transfected GBM#021 cell-derived xenografts at indicated times. **F** Kaplan–Meier survival analysis performed with survival data from indicated groups. ns: not significant, ∗: *p* < 0.05, ∗∗: *p* < 0.01, ∗∗∗: *p* < 0.001, and ∗∗∗∗: *p* < 0.0001

We also investigated the function of TRIM25 in vivo. Luciferase-expressing TRIM25-knockdown and control cell lines were orthotopically implanted in nude mice to monitor tumor growth (Fig. 3A). Based on the knockdown efficiency of TRIM25, we used sh-TRIM25-2-Luc for LN229, and sh-TRIM25-1-Luc for U251 (Supplementary Fig. S3A). At 28 days after implantation, tumor size was significantly smaller in TRIM25-knockdown groups compared to control groups (Fig. 3B, C). Kaplan–Meier survival analysis revealed a significantly extended survival in TRIM25-knockdown groups compared to control groups (median survival: 50 vs. 64 days for LN229; 39 vs. 68 days for U251; Fig. 3D). In order to comprehensively elucidate the role of TRIM25 in vivo, we used GBM#021 cells transfected with sh-NC-Luc/sh-TRIM25-2-Luc, selected based on the knockdown efficiency of TRIM25 (Supplementary Fig. S3A). Given the rapid growth of GBM#021 cells in mice, we conducted fluorescence live imaging of the mice at 5, 10, and 20 days after tumor implantation. The results showed significantly larger tumors in the control group compared to the TRIM25-knockdown group (Fig. 3E). Moreover, survival was shorter in the control group than in the TRIM25-knockdown group (median survival: 26 vs. 38 days for GBM#021; Fig. 3F). Collectively, these results indicated that the contribution of TRIM25 in promoting invasion and proliferation of GBM cells, both in vitro and in vivo.

### NONO is a potential interaction partner for TRIM25 in GBM cells

**Fig. 4:**
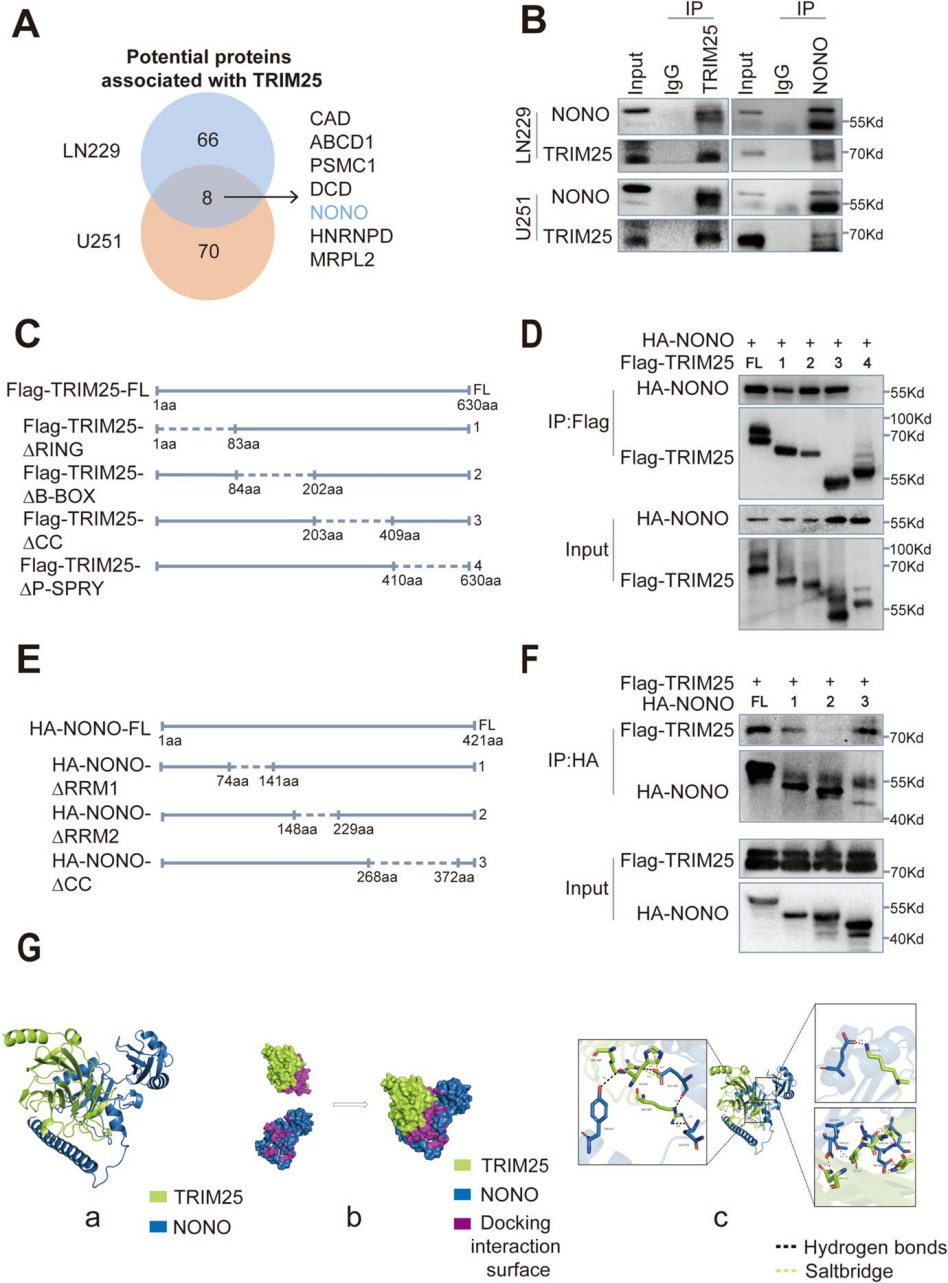
NONO is a potential interaction partner for TRIM25 in GBM cells. **A** Venn diagram of proteins that could potentially interact with TRIM25 in LN229 and U251 cells; After excluding proteins identified in the IgG groups, a total of eight proteins were found to interact with TRIM25 in both LN229 (66 proteins) and U251 (70 proteins). **B** Western blotting analysis of co-IP assays performed using anti-TRIM25/anti-NONO antibody and IgG antibody, with lysates prepared from LN229 and U251 cells. **C** Schematic representation of full-length (FL) TRIM25 and the following four deletion mutants: amino acids 1–83 (∆RING), 84–202 (∆B-BOX), 203–409 (∆CC), and 410–630 (∆P-SPRY); CC: coiled-coil. **D** Western blotting analysis of co-IP assays performed on lysates prepared from HEK293 cells transfected with HA-NONO and indicated Flag-TRIM25 constructs. **E** Schematic representation of full-length (FL) NONO and the following three deletion mutants: amino acids 74–141 (∆RRM1), 148–229 (∆RRM2), 268–372 (∆CC); CC: coiled-coil. **F** Western blotting analysis of co-IP assays performed on lysates prepared from HEK293 cells transfected with Flag-TRIM25 and indicated HA-NONO constructs. **G** Protein-protein docking visualization (green for TRIM25, blue for NONO). **a** Protein-protein docking based on a hybrid algorithm of template-based modeling and ab initio free docking. **b** Docking interaction surfaces of TRIM25 and NONO (indicated in purple). **c** Microscopic diagram showing mechanism of TRIM25-NONO interaction

To identify proteins potentially associated with TRIM25 in vivo, magnetic bead–Ab–Ag complexes were immunoprecipitated from LN229 and U251 cells using a TRIM25-specific antibody. Subsequently, the complexes were subjected to liquid chromatography-tandem mass spectrometry analysis. The results indicated that TRIM25 has the potential to interact with multiple proteins in both LN229 and U251 cells, primarily implicated in the regulation of chromosomes and RNA in the nucleus (Fig. 4A, Supplementary Fig. S4A). Notably, eight proteins were identified as shared between the two cell lines (Fig. 4A).

We selected NONO for further analysis based on previous research findings. As an oncogene, NONO is involved in nearly all steps of gene regulation and plays an important role in diverse tumor types [30–32]. One study demonstrated that NONO can enhance the pre-mRNA splicing process, thereby promoting proliferation and invasion of GBM cells [33]. To further explore the interaction between NONO and TRIM25, we performed co-IP assays. In both LN229 and U251 cells, NONO and TRIM25 were identified within the complexes immunoprecipitated from TRIM25 and NONO, respectively (Fig. 4B). To investigate the functional domains involved in their interaction, we designed a series of deletion mutant constructs for both TRIM25 and NONO (Fig. 4C and E). We transfected TRIM25 mutants and HA-NONO into HEK293 cells and subsequent co-IP assays showed that the deletion mutant of P-SPRY domain (amino acids 410–630) in TRIM25 exhibited diminished binding efficiency with NONO (Fig. 4D). In a parallel experiment, NONO mutants and Flag-TRIM25 were transfected into HEK293 cells and co-IP indicating that the deletion mutant of the RRM2 domain (amino acids 148–229) in NONO exhibited diminished binding efficiency with TRIM25 (Fig. 4F). The three-dimensional structures of TRIM25 and NONO (PDB ID: 6FLM, PDB ID: 3SDE) were obtained from the Protein Data Bank (PDB). Subsequent protein-protein docking simulations and visualization were conducted using HDOCK (http://hdock.phys.hust.edu.cn/). Consistent with the above results, these analyses showed that amino acids 435–630 in TRIM25 constitute the key structural domain for binding to NONO (docking score: -249.12, confidence score: 0.8789) (Fig. 4G, Supplementary Fig. S5A). In summary, our findings indicate that NONO serves as an interaction partner for TRIM25 in GBM. Furthermore, the P-SPRY domain (amino acids 410–630) of TRIM25 and the RRM2 domain (amino acids 148–229) of NONO are key structural domains governing the interaction between TRIM25 and NONO.

### TRIM25 promotes K63-linked ubiquitination of NONO

**Fig. 5:**
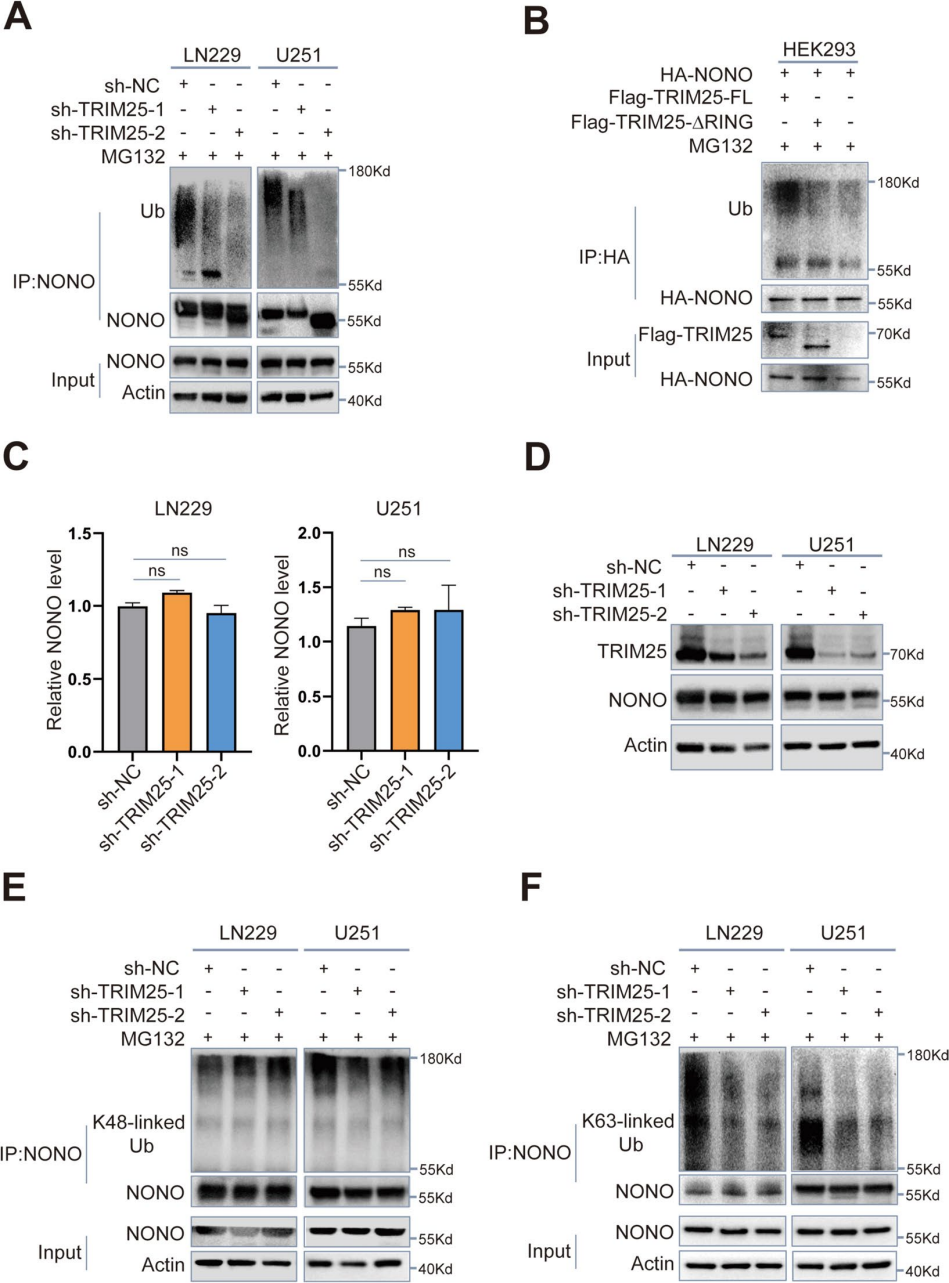
TRIM25 promotes K63-linked ubiquitination of NONO. **A** Western blotting analysis of NONO ubiquitination levels in the indicated transfected LN229 and U251 cells. **B** Western blotting analysis of HA-NONO ubiquitination levels in the indicated transfected HEK293 cells. **C** qRT-PCR analysis of NONO mRNA levels in transfected LN229 and U251 cells, relative to controls. **D** Western blotting analysis of NONO protein levels in TRIM25-knockdown cells. **E** Western blotting analysis of NONO via co-IP assays using K48-linked specific polyubiquitin antibody in the indicated transfected LN229 and U251 cells. **F** Western blotting analysis of NONO via co-IP assays using K63-linked specific polyubiquitin antibody in the indicated transfected LN229 and U251 cells

We hypothesized that, as a ubiquitin E3 ligase, TRIM25 could facilitate the ubiquitination of NONO, leading to degradation and functional alterations. To test this hypothesis, we evaluated the ubiquitination levels of NONO in TRIM25-knockdown LN229 and U251 cells subjected to a 6-hour treatment with the proteasome inhibitor MG132 (20 µM). Remarkably, cells with low TRIM25 expression exhibited a substantial reduction in ubiquitination levels (Fig. 5A). Given that the RING domain is pivotal for TRIM25’s role as an E3 ubiquitin ligase [20], we transfected HEK293 cells with HA-NONO along with either the RING domain deletion mutant of TRIM25 (Flag-TRIM25-∆RING) or the full length of TRIM25 (Flag-TRIM25-FL). Following treatment with MG132 (20 µM) for 6 h, the ubiquitination level of HA-NONO was significantly increseaed in the Flag-TRIM25-FL overexpression group, while the RING domain deletion mutant had no effect (Fig. 5B). Taken together, these findings suggested that the TRIM25 could promote the ubiquitination of NONO.

To further characterize the regulatory relationship between TRIM25 and NONO, we conducted qRT-PCR and Western blotting experiments on TRIM25-knockdown LN229 and U251 cells, as well as their corresponding controls. The results showed that TRIM25 expression exerted no influence on the mRNA and protein levels of NONO (Fig. 5C, D). This observation implies that TRIM25 is not involved in the regulation of GBM proliferation and invasion through modulation of NONO expression. Subsequent co-IP assays demonstrated that the level of K63-linked ubiquitination of NONO was considerably reduced after TRIM25 knockdown. However, the level of K48-linked ubiquitination only displayed slight alteration (Fig. 5E, F). Taken together, these results suggest that TRIM25 primarily regulates the level of K63-linked ubiquitination in NONO, rather than K48-linked ubiquitination.

### TRIM25-mediated K63-linked ubiquitination may regulate the splicing function of NONO, thereby affecting oncogenic c-MYC expression through PRMT1

**Fig. 6:**
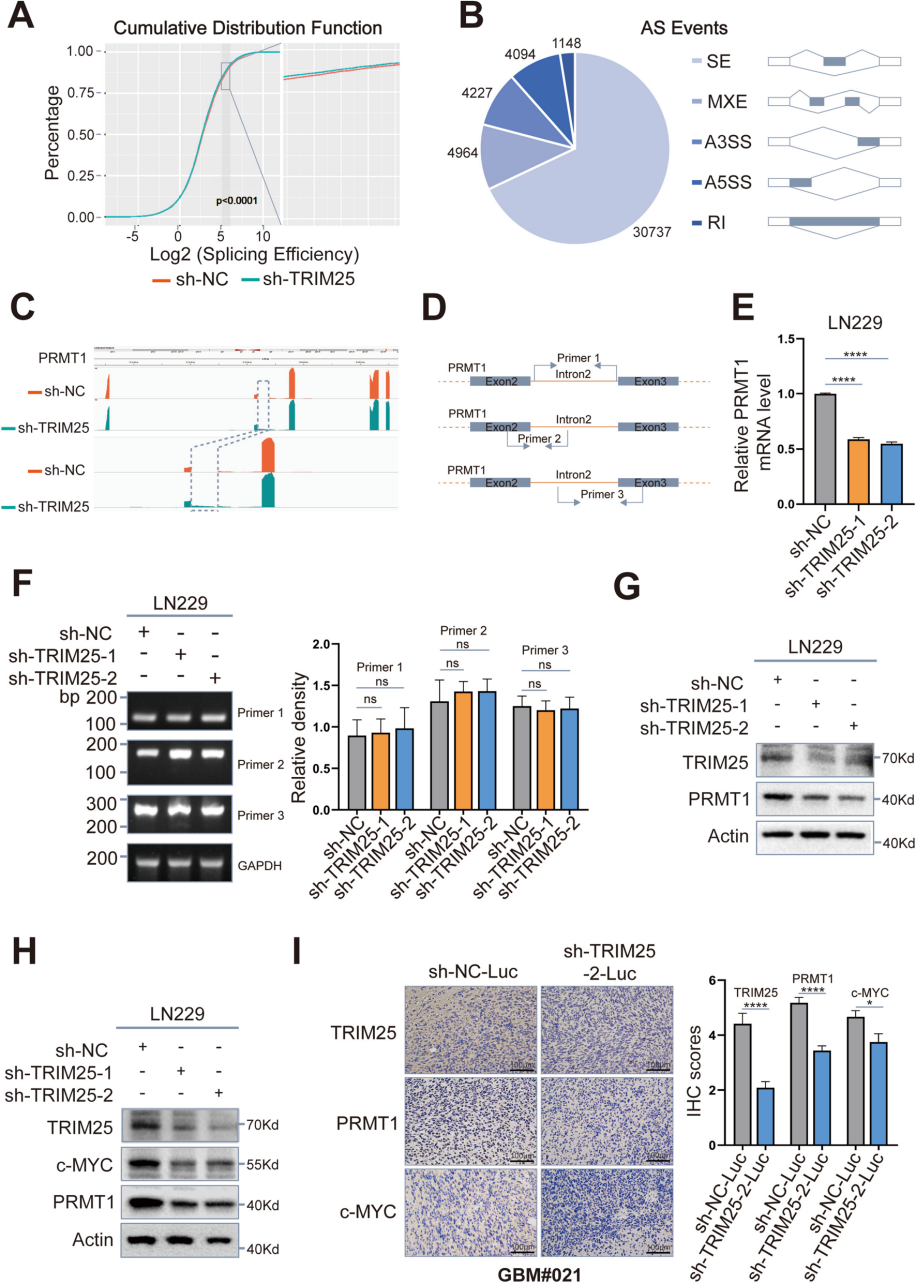
The dysregulated splicing function of NONO affects c-MYC expression through PRMT1. **A** Global splicing efficiency of TRIM25-knockdown LN229 cells and controls. Splicing efficiency was calculated as follows: splicing efficiency = transread count / 5’ and 3’ intron end first base coverage. **B** Quantification of alternative splicing (AS) events after TRIM25 knockdown in LN229 cells. AS events are classified into five categories: skipped exon (SE), mutually exclusive exons (MXE), alternative 5’ splice site (A5SS), alternative 3’ splice site (A3SS), and retained intron (RI). **C** The depths of mapped reads for different genomic regions of PRMT1 were visualized by IGV. The location of intron 2 is indicated by the dashed box. **D** The schematic representation of primers for the retained intron 2 in pre-mRNA of PRMT1. **E** qRT-PCR analysis of mature PRMT1 mRNA levels in TRIM25-knockdown LN229 cells, relative to controls. **F** qRT-PCR analysis of the retained intron 2 in pre-mRNA of PRMT1, GAPDH was used for normalization. **G** Western blotting analysis of PRMT1 protein levels in TRIM25-knockdown and control LN229 cells. **H** Western blotting analysis of c-MYC protein levels in TRIM25-knockdown and control LN229 cells. **I** Representative images of IHC for TRIM25, PRMT1, c-MYC in sections from intracranial xenografts derived from GBM#021-NC and -sh-TRIM25 implanted in nude mice, scale bar = 100 μm. ns: not significant, ∗: *p* < 0.05, ∗∗: *p* < 0.01, ∗∗∗: *p* < 0.001, and ∗∗∗∗: *p* < 0.0001

K63-linked ubiquitination is an important type of post-translational modification that regulates protein interaction, translocation, and activation [34, 35]. Previous research findings suggest that K63-linked ubiquitination affects mRNA splicing, either through direct regulation of the splicing function of the spliceosomal protein PRP31 [36] or by regulating the assembly of specific spliceosomal complexes [37, 38]. Therefore, we hypothesized that TRIM25-mediated K63-linked ubiquitination of NONO could impact the splicing function of NONO. To test this hypothesis, we performed high-throughput RNA-Seq of TRIM25-knockdown and control LN229 cells. The results indicated a significant reduction in global splicing efficiency in TRIM25-knockdown LN229 cells compared to control LN229 cells (Fig. 6A). Furthermore, compared with the control group, tens of thousands more NONO-mediated alternative splicing (AS) events were identified in the TRIM25-knockdown group. These events were categorized into five AS types, as illustrated in Fig. 6B, with skipped exon (SE) events constituting the majority.

Previous reports indicated that PRMT1 is involved in the development of many tumors [39–41]. Moreover, PRMT1 was an insufficient splicing gene identified by the analysis of RNA-Seq results using the R package *SEAA*. IGV-based visualization showed a notable increase in the retention of the second intron in PRMT1 mRNA (Fig. 6C). To confirm this intron retention, we designed specific primers targeting the second intron in the pre-mRNA of PRMT1 (Fig. 6D, Supplementary Table S4) and the mature mRNA of PRMT1 (Supplementary Table S44). Although the relative levels of mature mRNA in TRIM25-knockdown LN229 cells were significantly decreased compared with the controls (Fig. 6E), the levels of the retained intron remained constant or slightly increased (Fig. 6F). These results indicated that the PRMT1 pre-mRNA was insufficiently spliced and resulted in intron retention. The expression of the PRMT1 protein was also evaluated. Western blotting results revealed a significant decrease in PRMT1 expression in TRIM25-knockdown groups compared to controls (Fig. 6G). As an oncogene, PRMT1 is known to regulates the protein level of c-MYC by promoting its transcription and maintaining its stability [42, 43]. Western blotting experiments revealed a significant reduction in the protein level of c-MYC in TRIM25-knockdown groups compared to corresponding controls (Fig. 6H). Immunohistochemical staining of sections from tumor xenograft models demonstrated reduced protein levels of PRMT1 and c-MYC in tumor xenografts derived from TRIM25-knockdown GBM#021 cells relative to controls (Fig. 6I). These results suggest that the inhibition of K63-linked ubiquitination of NONO via TRIM25 knockdown could suppress the splicing function of NONO, resulting in the retention of the second intron in the pre-mRNA of PRMT1 and subsequent downregulation of c-MYC.

## Discussion

**Fig. 7:**
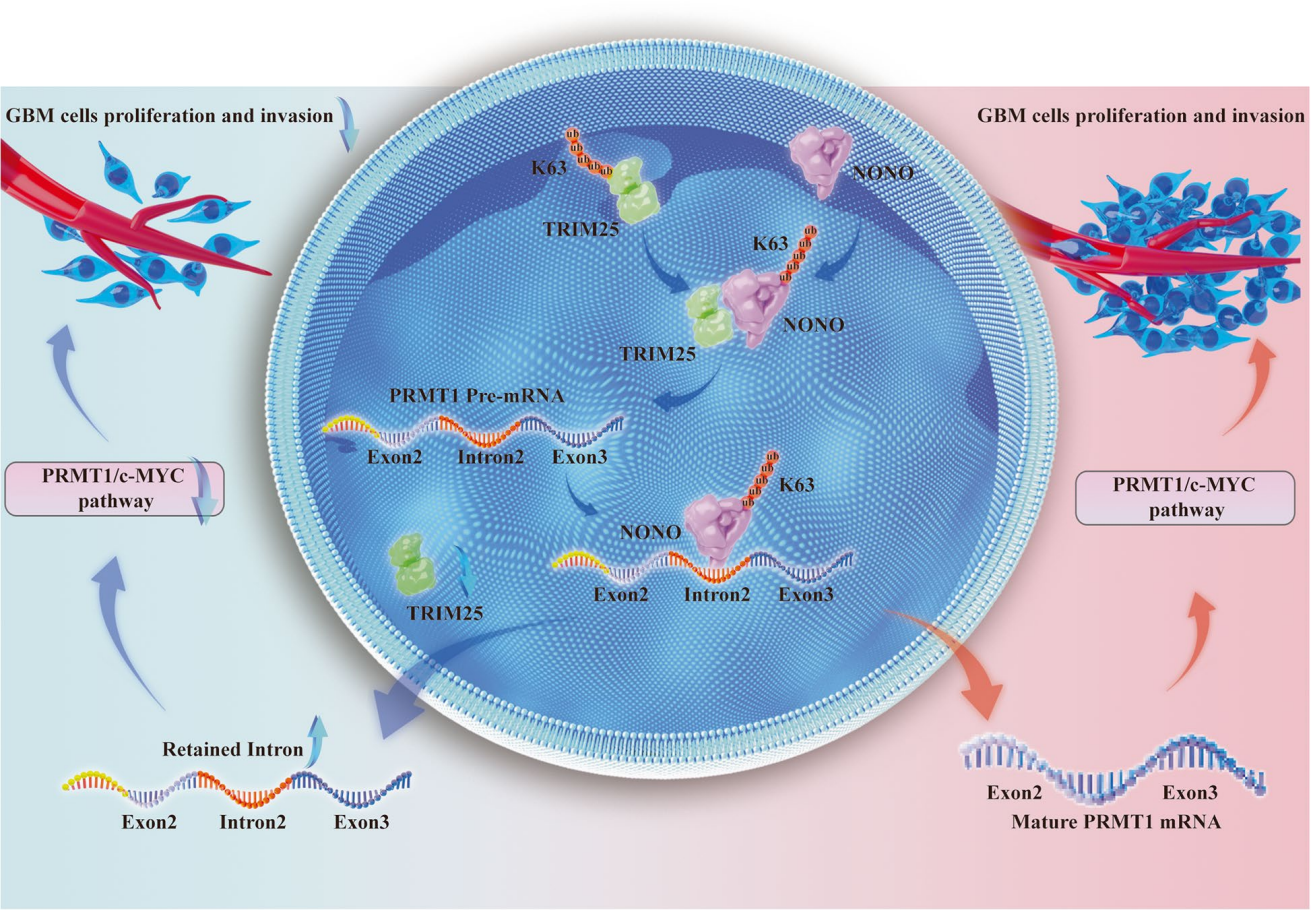
Schematic model illustrating the role of TRIM25 in glioblastoma cell growth and invasion. TRIM25 mediates the K63-linked ubiquitination of NONO and maintains normal physiological functions of NONO in GBM cells. TRIM25 knockdown reduces the K63-linked ubiquitination of NONO, which inhibits the splicing function of NONO. The dysfunctional NONO further leads to the retention of the second intron in the pre-mRNA of PRMT1 and suppresses the production of mature PRMT1 mRNA, thereby inhibiting the activation of the c-MYC pathway

The ubiquitination process is involved in various biological responses, including cell growth, DNA damage repair, and signal transduction. As the most specialized enzymes within the ubiquitination system, E3 ligases are considered promising drug targets for human diseases such as cancer. Thus, patients with GBM may benefit from the inhibition of specific E3 ligases, rather than the general proteasome inhibitor bortezomib. In this study, we found that TRIM25 is upregulated in glioma and promotes tumor growth. We subsequently identified NONO as a potential substrate of TRIM25. Knockdown of TRIM25 suppressed NONO’s splicing function by reducing K63-linked ubiquitination, thereby disruptiing the activation of the PRMT1/c-MYC pathway (Fig. 7).

TRIM25, a member of the TRIM family of proteins, is preferentially expressed in various human cancers, including breast, ovarian, lung, and gastric cancers [44, 45]. This study revealed a significant upregulation of TRIM25 in GBM, correlating with a poor prognosis. The oncogenic role of TRIM25 was validated both in vitro and in vivo. As an E3 ubiquitin ligase, TRIM25 needs interact with its substrate to promote tumor growth. TRIM25 can prevent cell necrosis by directly interacting with and ubiquitinating RIP3 [46]. In hepatocellular carcinoma, TRIM25 regulates endoplasmic reticulum homeostasis and mitigates oxidative stress by mediating the ubiquitination and degradation of Keap1 [24]. Furthermore, TRIM25 interacts with G3BP2 to modulate P53 signals, promoting the growth and survival of prostate cancer cells [28]. The present study revealed that TRIM25 could regulate the ubiquitination of NONO, and affect pre-RNA splicing efficiency by promoting the K63-linked ubiquitination of NONO. Taken together, recent evidence suggests that TRIM25 play a multifaceted role in tumor cells via substrate modification, particularly ubiquitination. In addition, we found that knockdown of TRIM25 with different sequences resulted in different inhibitory effects on the ubiquitination level of NONO (Fig. 5A), indicating that the mechanism of TRIM25-mediated ubiquitination of NONO is complex and further molecular biology studies are needed to clarify this issue.

Ubiquitination is an important post-transcriptional modification of proteins that plays important roles in nearly all aspects of tumor biology, including proliferation, invasion, metastasis, microenvironment, recurrence, and drug resistance [47–49]. The diversity of ubiquitin linkage types (K6, K11, K27, K29, K33, K48, and K63) is a key feature of ubiquitination, enabling the activation of diverse signaling pathways and dictates the fate of substrate proteins. In recent years, advances in K63 research have demonstrated that ubiquitination is not solely involved in protein degradation [12, 50, 51]. In glioblastoma, K63-linked ubiquitination can modulate the activities, interactions, or trafficking of tagged proteins pivotal to diverse various oncogenic processes. For example, Nedd4-1 can induce K63-linked ubiquitination of Rap2a in glioma, leading to inhibition of GTP-Rap2a activity [52]. F-Box and FBXL18 can activate PI3K-Akt signaling in glioma cells by promoting the K63-linked ubiquitination of Akt [53]. In glioblastoma, Nrdp1-mediated K63-linked polyubiquitination inhibits the function of Dvl (a Wnt pathway protein) by preventing its association with the plasma membrane [54]. These prompt speculation that K63-linked ubiquitination plays a role in regulating the splicing function of target proteins. This may occur by the direct inhibition of splicing factors or by blocking protein-protein interactions in specific spliceosomes. Indeed, ubiquitination is required to regulate the assembly of the U4/U6.U5 tri-snRNP complex by PRP8-ubiquitin conjugation [38]. K63-linked ubiquitination of U4 snRNP components, mediated by the PRP19 complex, stabilizes the U4/U6.U5 tri-snRNP complex, thereby promoting spliceosome activation [36, 37]. Moreover, the reversible ubiquitination of these spliceosomes implies that their splicing efficiency is subject to influence by multiple factors through a mechanism that requires further investigation.

NONO (p54nrb) was first described in 1993 as a 54-kDa nuclear RNA-binding protein that promotes pre-mRNA processing [55]. In recent years, mounting evidence suggests that NONO participates in tumorigenesis by regulating proliferation, apoptosis, cell migration, and DNA damage repair [30]. In hepatocellular carcinoma, NONO is implicated in the production of a long isoform of bridging integrator 1 (BIN1) through exon 12a inclusion, contributing to enhanced malignancy [56]. Moreover, NONO promotes prostate cancer growth by inducing aberrant RNA splicing of EPHA6, resulting in the truncated EPHA6-001 variant. In addition to the effects of NONO on exons, NONO knockdown induces intron retention in GPX1 pre-mRNA, thereby impeding GBM progression [33]. Overall, the pre-RNA splicing ability of NONO is important for its participation in the tumor growth process.

Pre-mRNA splicing is required for the maturation of nearly all human mRNAs. Emerging evidence that aberrant AS events are involved in tumor initiation and/or development [57, 58]. Recent RNA-Seq analyses of 16 tumor types and paired normal tissues revealed that skipped exons constituted 50–60% of all AS events, whereas retained introns constituted fewer than 20% of AS events in all cancers and normal tissues, consistent with the pattern observed in our study [59]. Intriguingly, transcriptome analyses of various cancer types often identify significant increases in intron retention compared to adjacent normal tissues [59]. Consequently, the immature RNAs induced by retained introns or malfunctioning proteins translated from intron-retaining mRNAs warrant further investigation in cancer research. In tthis study, we found that the second intron was retained in the pre-mRNA of PRMT1 after TRIM25 knockdown. This retention subsequently led to a significant reduction in the mature mRNA of PRMT1. Generally, retained introns harboring a premature termination codon can trigger the nonsense-mediated decay pathway, leading to the degradation of RNAs containing such retained introns and consequently diminishing the expression of specific proteins [60, 61]. Notably, a retained intron in EIF2B5 contained a premature termination codon impedes global translation, allowing cancer cells to adapt to a hypoxic environment [62]. However, certain intron-retaining mRNAs do not activate nonsense-mediated decay; instead, they serve distinct functions in the organism. For instance, Kallikrein genes retaining intron III lose enzymatic activity in prostate cancer, and the intron III-retaining kallikrein-15 variant emerges as a potential biomarker for prostate cancer [63]. The inhibition of intron excision by RNA-targeting thiomorpholino antisense oligonucleotides enhances the nuclear compartmentalization of TERT mRNA and TUG1 lncRNA, highlighting the role of retained introns in RNA localization [64]. However, the available evidence does not conclusively determine the fate of intron-retaining mRNAs, necessitating further investigation into the underlying mechanisms.

## Conclusion

In this study, we uncovered a novel regulatory signaling axis that supports GBM progression. TRIM25 promotes K63-linked ubiquitination of NONO to enhance splicing efficiency. TRIM25 knockdown leads to the retention of the second intron in the pre-mRNA of PRMT1, impeding the production of mature PRMT1 mRNA and subsequently suppressing the activation of the c-MYC pathway in GBM cells. Targeting the E3 ligase activity of TRIM25 and disrupting the TRIM25-NONO interaction emerges as a promising therapeutic strategy for GBM treatment.

### Supplementary Information

**Additional file 1.** 

## Data Availability

The datasets used and/or analysed during the current study are available from the corresponding author on reasonable request.

## Abbreviations

GBM: Glioblastoma Multiforme
TRIM: Tripartite Motif
TRIM25: Tripartite Motif 25
RIG-I: Retinoic Acid-Inducible Gene I
Keap1-Nrf2: Kelch-Like ECH-Associated Protein 1–NF-E2 P45-Related Factor
MAP3K13: Mitogen-Activated Protein Kinase Kinase Kinase 13
G3BP2: Granule Assembly Factor 2
PRMT1: Protein Arginine Methyltransferase 1
GSCs: GBM Stem-Like Cells
HEK293: Human Embryonic Kidney Cell Line 293
IHC: Immunohistochemistry
WB: Western Blotting
qRT-PCR: Real-Time Quantitative RT-PCR
Co-IP: Co-Immunoprecipitation
TCGA: The Cancer Genome Atlas
CGGA: The Chinese Glioma Genome Atlas
GEO: Gene Expression Omnibus
IGV: Integrative Genomics Viewer
SEAA: Splicing Efficiency Analysis and Annotation
AS: Alternative Splicing
SE: Skipped Exon
MXE: Mutually Exclusive Exons
A5SS: Alternative 5’ Splice Site
A3SS: Alternative 3’ Splice Site
RI: Retained Intron
RIP3: Receptor-Interacting Protein Kinase 3
GTP: Guanosine Triphosphate
FBXL18: Rich Repeat Protein 18
PI3K- Akt: Phosphatidylinositol 3-Kinase–Protein Kinase B
BIN1: Bridging Integrator 1
TERT: Telomerase Reverse Transcriptase
LncRNA: Long Noncoding RNA
TUG1: Taurine Up-Regulated 1
NONO: Non-POU Domain Containing Octamer Binding
SEM: Standard Error of The Mean
FL: Full-Length
