# Supplementary material for: TRIM25 promotes glioblastoma cell growth and invasion via regulation of the PRMT1/c-MYC pathway by targeting the splicing factor NONO
Source: J Exp Clin Cancer Res. 2024 Feb 2;43:39. doi: 10.1186/s13046-024-02964-6 (PMC10835844; doi:10.1186/s13046-024-02964-6)

**Supplementary Figure Legends**

**Supplementary Fig. S1 A.** Images of TRIM25 IHC assays performed on WHO grade II–IV glioma tissues and nonneoplastic brain tissues (NBTs). Scale bar = 100 μm (top). **B.** Western blotting analysis of TRIM25 protein levels in primary glioma tissues (n = 10) and NBT (n = 1).

**Supplementary Fig. S2 A.** Representative images of colony-forming assays of transfected LN229 and U251 cells. Data are presented as mean ± SEM in bar graphs. **B**. Growth curves for TRIM25-knockdown GBM#021 cells generated by cell counting over 72 hours.

**Supplementary Fig. S3 A.** Western blotting results for the knockdown efficiency of TRIM25 in the transfected LN229, U251 and GBM#021 cells used for the orthotopic glioma model.

**Supplementary** **Fig. S4 A.** Bubble graphs show Gene Ontology (GO) and Kyoto Encyclopedia of Genes and Genomes (KEGG) enrichment analysis of potential interaction partners for TRIM25 in LN229 and U251 cells.

**Supplementary Fig. S5 A.** The 2D protein interaction diagram was generated using Ligplot software, with Chain A representing TRIM25 and Chain B representing NONO.

**Supplementary Table S1** Sequences of shRNAs and siRNAs used in this study.

**Supplementary Table S2** Information of plasmid used in this study.

**Supplementary Table S3** Antibodies used for Western blotting and co-IP.

**Supplementary Table S4** Primer sets used in this study.

**Supplementary Table S1**

**Sequences of shRNAs and siRNAs used in this study**

| si-NC | 5’-UUCUCCGAACGUGUCACGUTT-3’ |
| --- | --- |
| si-TRIM25-1 | 5’-UCCUGGAGUAUUACAUUAATT-3’ |
| si-TRIM25-2 | 5’-UCUGUCAGAGUGCUAUACATT-3’ |
| sh-NC | 5’-CCTAAGGTTAAGTCGCCCTCG-3’ |
| sh-TRIM25-1 | 5’-GCTTTCGAGACGATGATTATT-3’ |
| sh-TRIM25-2 | 5’-AGGATGAGGTCGGGTACATAT-3’ |

**Supplementary Table S2**

**Plasmid information and sources**

| pcDNA3.1(+)-NONO-HA | OBiO Technology |
| --- | --- |
| pcDNA3.1(+)-NONO(∆74-141aa)-HA | OBiO Technology |
| pcDNA3.1(+)-NONO(∆148-229aa)-HA | OBiO Technology |
| pcDNA3.1(+)-NONO(∆268-372aa)-HA | OBiO Technology |
| pcDNA3.1(+)-empty vector-HA | OBiO Technology |
| pcDNA3.1(+)-TRIM25-3×FLAG | OBiO Technology |
| pcDNA3.1(+)-TRIM25(∆1-83aa)-3×FLAG | OBiO Technology |
| pcDNA3.1(+)-TRIM25(∆84-202aa)-3×FLAG | OBiO Technology |
| pcDNA3.1(+)-TRIM25(∆203-409aa)-3×FLAG | OBiO Technology |
| pcDNA3.1(+)-TRIM25(∆410-630aa)-3×FLAG | OBiO Technology |
| pcDNA3.1(+)-empty vector-3×FLAG | OBiO Technology |

**Supplementary Table S3**

**Antibodies used for Western blotting and co-IP**

| TRIM25 | Proteintech (67314-1-Ig) | WB: 1:5000 |
| --- | --- | --- |
| TRIM25 | Proteintech (12573-1-AP) | WB: 1:1000, IP: 1-10 μg |
| NONO | Proteintech (11058-1-AP) | WB: 1:1000, IP: 1-10 μg |
| NONO | Proteintech (66361-1-Ig) | WB: 1:1000 |
| HA-tag | Cell Signaling Technology (3724) | WB: 1:1000, IP: 1-10 μg |
| HA-tag | ABclonal (AE008) | WB: 1:2000 |
| Flag-tag | ABclonal (AE005) | WB: 1:2000 |
| Flag-tag | ABclonal (AE063) | WB: 1:2000, IP: 1-10 μg |
| c-MYC | Proteintech (10828-1-AP) | WB: 1:1000 |
| PRMT1 | Proteintech (11279-1-AP) | WB: 1:2000 |
| Ubiquitin | Thermo Fisher (14-6078-80) | WB: 1:250 |
| K48-linkage ubiquitin | Cell Signaling Technology (8081) | WB: 1:1000 |
| K63-linkage ubiquitin | Cell Signaling Technology (5621) | WB: 1:1000 |
| Beta-actin | Proteintech (66009-1-Ig) | WB: 1:1000 |
| IgG | Proteintech (30000-0-AP) | IP: 1-10 μg |

**Supplementary Table S3**

**Primer sets used in this study**

| NONO-F | CTCCTTCCTGCTAACCACATTTC |
| --- | --- |
| NONO-R | TTGATGTTGCGGTCCACTTG |
| PRMT1-Intron2-F (Primer 1) | CACCCTGACCTCCACATCAATA |
| PRMT1-Intron2-R (Primer 1) | AGGGGTTGCTACTAAGTGTCTGG |
| PRMT1-Intron2-F (Primer 2) | CCAGCCGCCTCTTGAAGAA |
| PRMT1-Intron2-R (Primer 2) | AGGGGTTGCTACTAAGTGTCTGG |
| PRMT1-Intron2-F (Primer 3) | CACCCTGACCTCCACATCAATA |
| PRMT1-Intron2-R (Primer 3) | CACGCCTACCATCTCTCCAGTC |
| PRMT1-F | GCACCCTCACTTACCGCAACT |
| PRMT1-R | TTTGACGATCTTCACCGCATAA |

**Fig. S1**


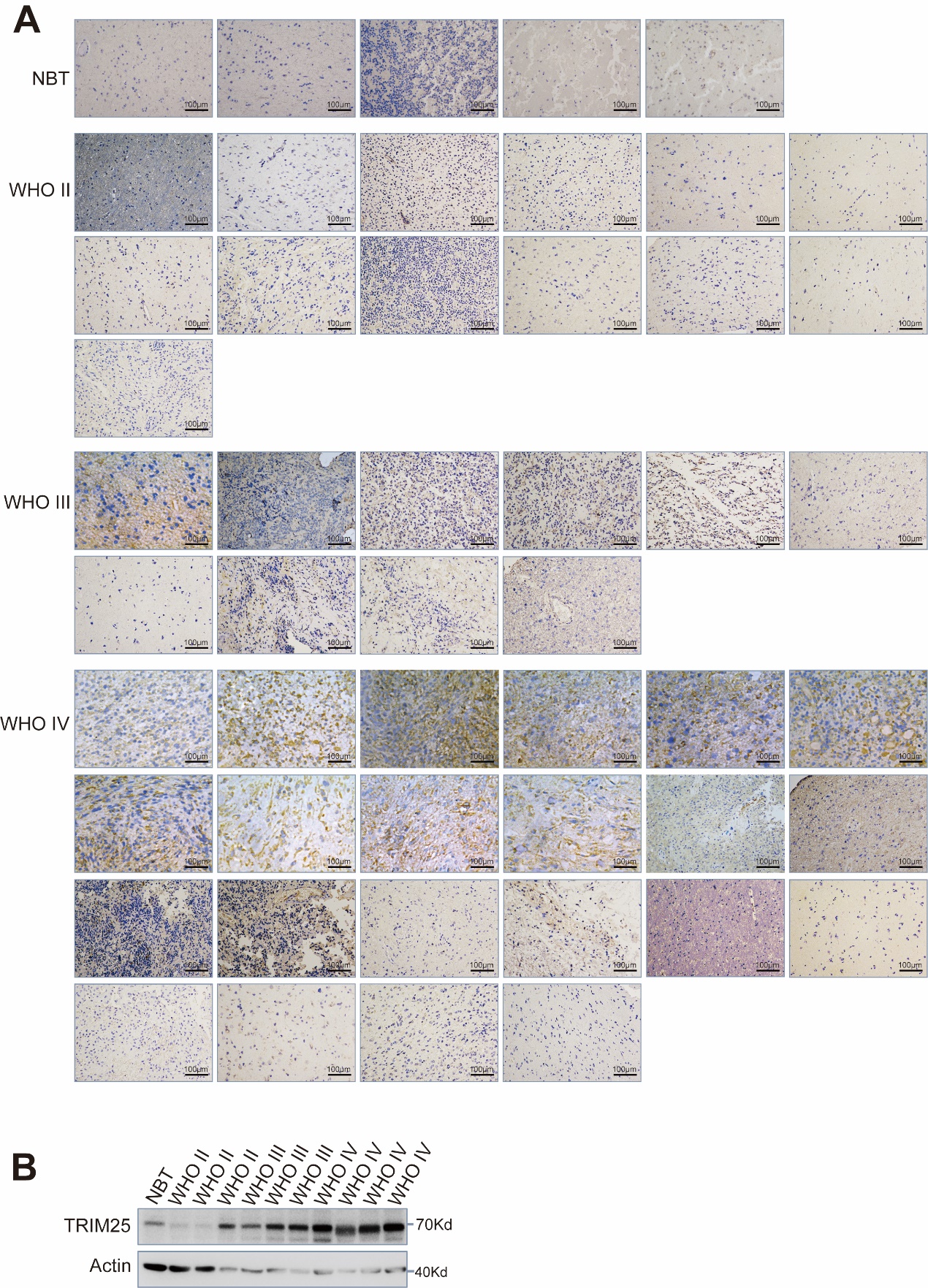


**Fig. S2**


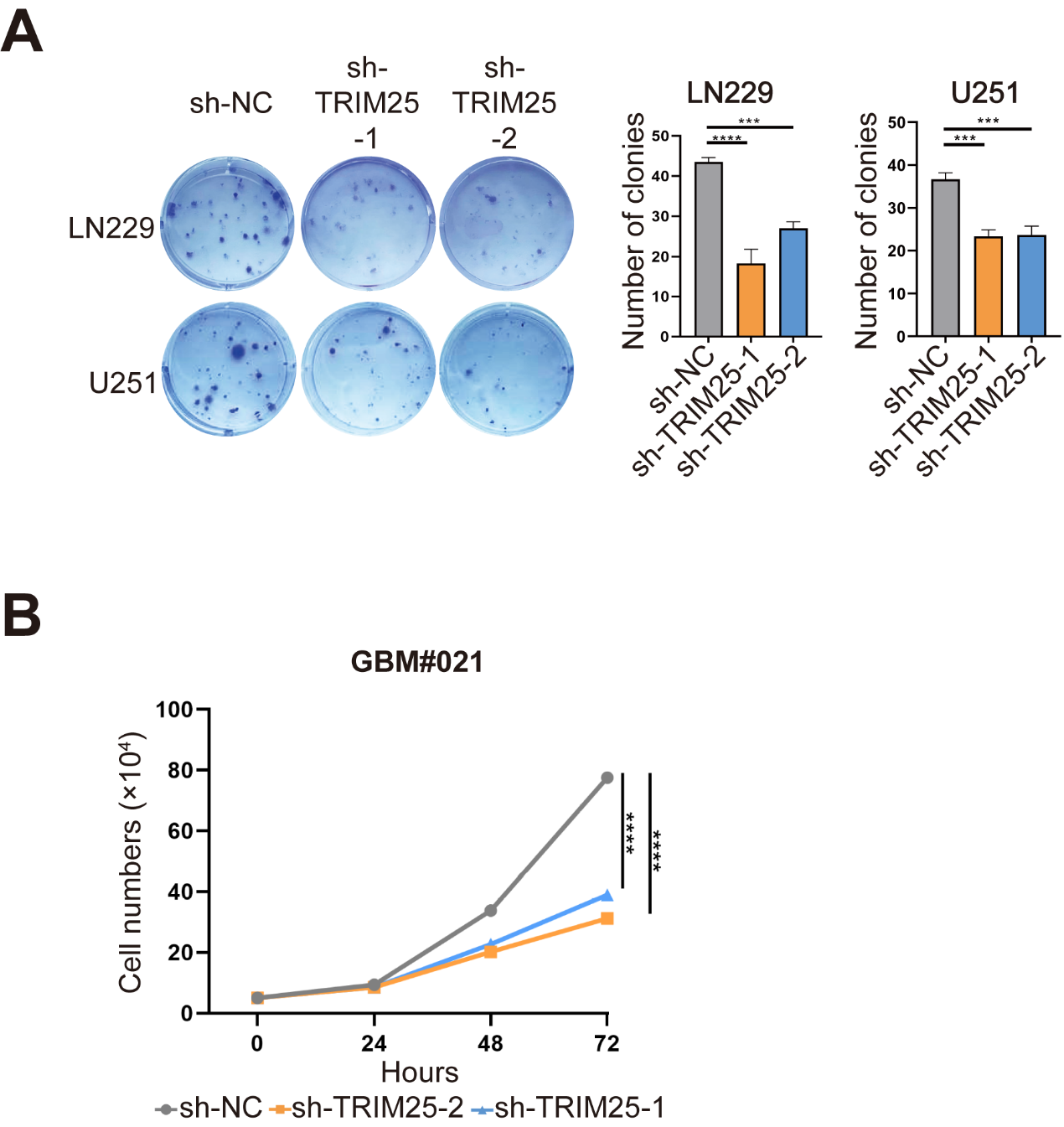


**Fig. S3**


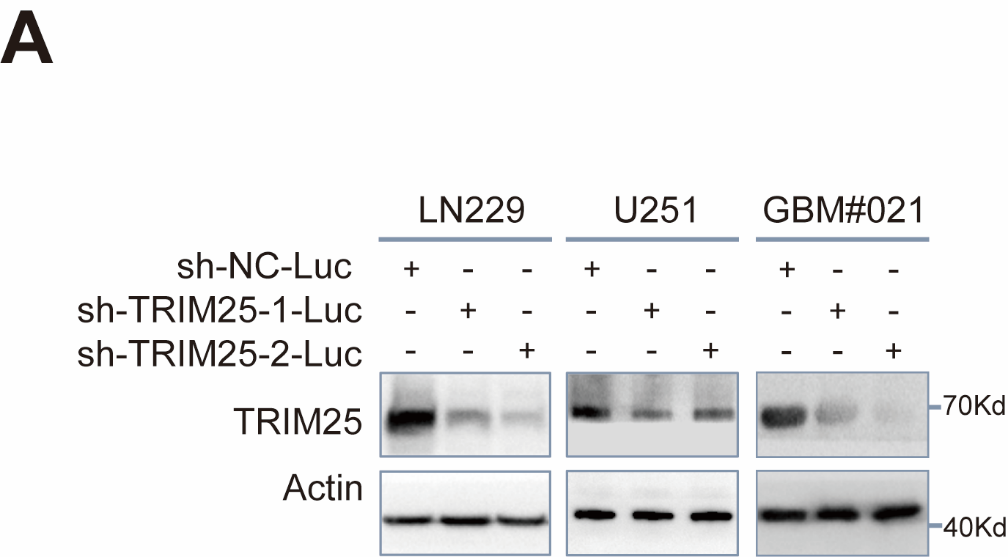


**Fig. S4**


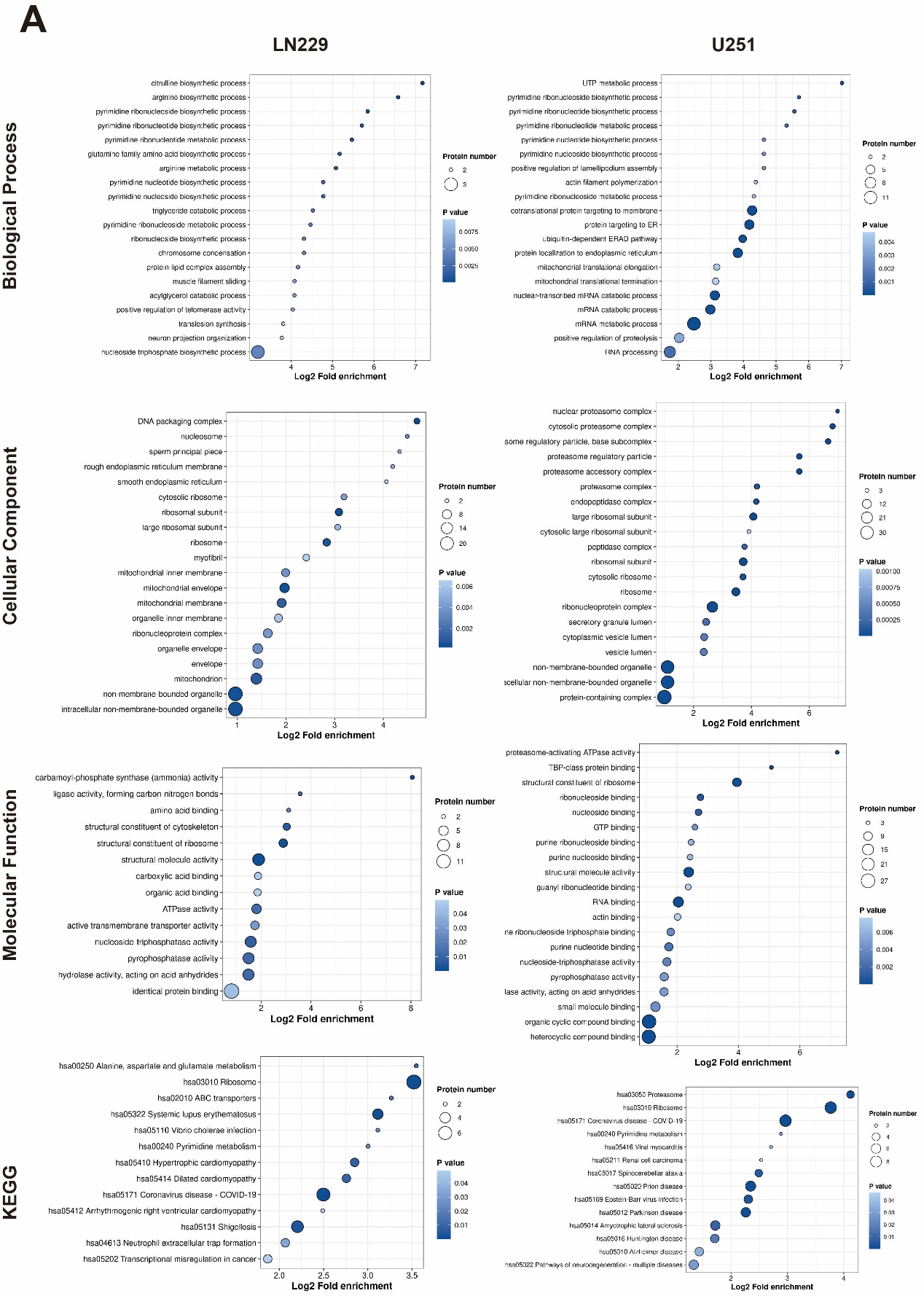


**Fig. S5**


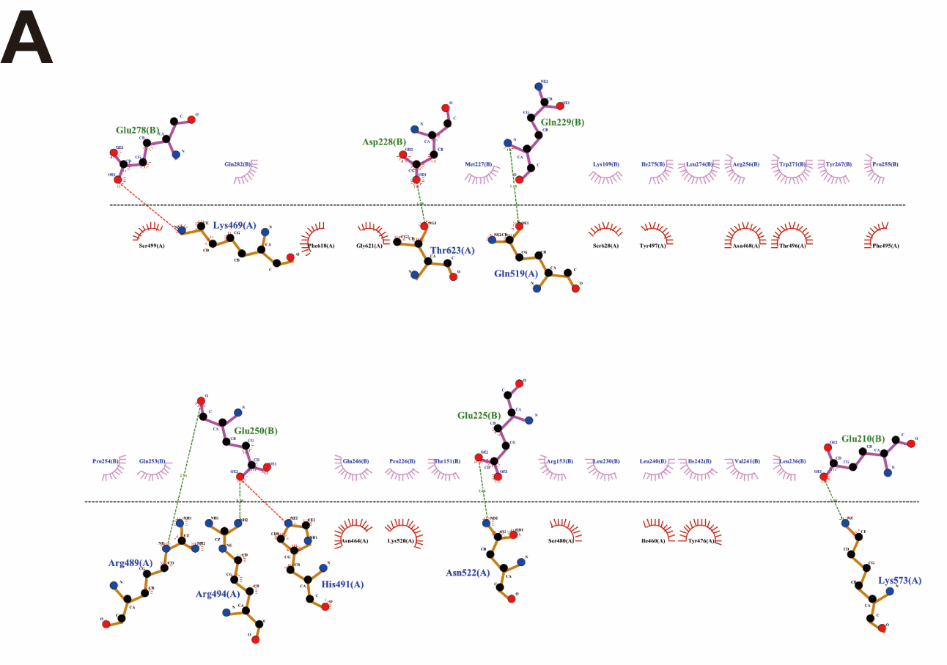

Supplement: Supplementary file 1 — Additional file 1. [file 13046_2024_2964_MOESM1_ESM.docx]
